# *Candida auris*: A Mini Review on Epidemiology in Healthcare Facilities in Asia

**DOI:** 10.3390/jof8111126

**Published:** 2022-10-26

**Authors:** Nishanthinie Thatchanamoorthy, Velayuthan Rukumani Devi, Samudi Chandramathi, Sun Tee Tay

**Affiliations:** 1 Department of Medical Microbiology, Faculty of Medicine, University of Malaya, Jalan Profesor Diraja Ungku Aziz, Kuala Lumpur 50603, Wilayah Persekutuan, Malaysia; 2 Department of Medical Microbiology, University Malaya Medical Centre, Jalan Profesor Diraja Ungku Aziz, Kuala Lumpur 59100, Wilayah Persekutuan, Malaysia

**Keywords:** *Candida auris*, epidemiology, invasive fungal infections

## Abstract

*Candida auris,* a newly emerging healthcare-associated yeast pathogen from the Metschnikowiaceae family, was first described in the ear canal of an elderly Japanese patient in 2009. The yeast is one of the causative agents of candidemia, which has been linked with nosocomial outbreaks and high mortality rates in healthcare facilities worldwide. Since its first isolation, the occurrence of *C. auris* in six continents has becomes a grave concern for the healthcare professionals and scientific community. Recent reports showed the identification of five geographically distinct clades and high rates of antifungal resistance associated with *C. auris*. Till date, there are no effective treatment options, and standardized measures for prevention and control of *C. auris* infection in healthcare facilities. This leads to frequent therapeutic failures and complicates the eradication of *C. auris* infection in healthcare facilities. Thus, this review focuses on the recent understanding of the epidemiology, risk factors, diagnosis, transmission and prevention and control strategies of *C. auris* infection in healthcare facilities in Asia.

## 1. Introduction

*Candida auris* is a newly emerging multidrug-resistant yeast pathogen notorious for causing nosocomial outbreaks in many healthcare facilities worldwide. High mortality rates of *C. auris* infection have been reported among critically ill patients [1]. Both pediatric and adult patients with prolonged intensive care unit (ICU) stay, who are previously exposed to broad-spectrum antibiotics, and undergo invasive medical procedures, are at high risk of *C. auris* infection [2]. *C. auris* was initially misidentified as other yeast species such as *Candida haemulonii*, *Candida famata*, *Candida sake*, *Rhodotorula glutinis*, and *Saccharomyces cerevisiae* when the pathogen was subjected to biochemical-based identification methods using Vitek 2, API ID32C, and Auxacolor commercial systems [3]. The introduction of internal transcribed spacer (ITS) and 28S ribosomal DNA (rDNA) gene sequencing approach, and matrix-assisted laser desorption ionization-time of flight spectrometry systems (MALDI-TOF MS) have provided more accurate identification and differentiation of *C. auris* from other *Candida* species [2]. Hospital surveillance studies showed that this opportunistic pathogen can be acquired from both dry and moist environmental surfaces in healthcare facilities [4]. The ability to survive for a long period on hospital surfaces have been linked with the production of various virulence factors including hydrolytic enzymes, and biofilm forming capability that aids persistent colonization of *C. auris* on human skin and the environmental surface [4]. Hence, more effective disinfectants and antiseptics are critically needed to enhance the implementation of hospital infection control measures [5].

## 2. Epidemiology

### 2.1. The Emergence of C. auris

*C. auris* is an ascomycetous yeast that was first identified and reported by Satoh et al. [6] as a novel species from the ear canal of an elderly inpatient at Tokyo Metropolitan Geriatric hospital, Japan. The sequences of the 26S rDNA D1/D2 domain and ITS region of the isolate showed only 85.7% and 87.5% similarity to *C. haemulonii,* respectively. Further analysis showed that the isolate was phylogenetically related to *Candida pseudohaemulonii*, *Candida heveicola*, and *Candida ruelliae*. Moreover, the biochemical analysis of the isolate showed distinct characteristics from other *Candida* species including unique carbon assimilation patterns and ability to grow at 42 °C, thus further confirming the identification of *C. auris* as a novel *Candida* species [7].

Additionally, analysis of 15,271 *Candida* isolates collected from Asia, Latin America, Europe, and North America revealed the presence of four *C. auris* isolates collected from 2009–2015, hence; supporting *C. auris* as an emergent human pathogen [8]. Whole-genome sequencing (WGS) had been conducted for 47 *C. auris* isolates collected from different geographical locations (i.e., India, Pakistan, Japan, Venezuela, and South Africa). The single-nucleotide polymorphism analysis of the whole genome sequences revealed four geographically distinct clades, which include Clade I (South Asian), Clade II (East Asian), Clade III (African), and Clade IV (South American). The single nucleotide polymorphisms (SNP) differences observed between the isolates from each clade suggest the dynamic evolution of the organism [9]. However, the isolates share low genetic variations within their geographic clade [10]. A newly emerged fifth *C. auris* clade with >200,000 SNP differences from other clades has been reported in Iran [11]. Interestingly, while the invasive infections, nosocomial transmission, and large-scale healthcare outbreaks were mainly caused by Clade I, III and IV; Clade II has been reported to cause ear infection so far [12].

### 2.2. Incidence of C. auris Infections in Asia (1996–2021)

Table 1 summarizes the incidences of *C. auris* infection in Asia from 1996–2021. Following the first isolation of *C. auris* in Japan (2009), *C. auris* infection including candidemia has been subsequently described by a number of countries in Asia. The first *C. auris* candidemia was reported from a retrospective analysis of South Korean unidentified *Candida* isolates using rDNA sequencing. The analysis revealed that *C. auris* was first isolated back in 1996 from the bloodstream infection of a one-year-old patient with hypoxic encephalopathy and aspiration pneumonia [13]. In China, Wang et al. [14] described the first case of *C. auris* infection from the bronchoalveolar lavage of an elderly patient in 2018. An additional seventeen cases were later documented in Beijing and Shenyang, China [15,16]. Except for a strain belonging to the South Asian clade which was susceptible to all antifungals, the remaining strains isolated from China were of the South African clade and fluconazole resistant [14,15,16]. A *C. auris* strain was later isolated in south China and found to be closely related to the Shenyang and Beijing isolates [17]. Meanwhile, *C. auris* was also reported to affect 15 patients in a hospital in Hong Kong, with all strains being identified as belonged to the South Asia clade by WGS analysis [18]. Tang et al. [19] reported the first isolation of *C. auris* from the ruptured vesicles of a diabetic patient in Taiwan.

In South Asia, a candidemia outbreak was first reported from a hospital in India [20]. *C. auris* was isolated from 15 patients of which 13 isolates were identified as *C. haemulonii* initially but later confirmed as *C. auris* through rDNA sequencing. The first candidemia outbreak was reported in Pakistan in 2015 [21]. The isolates were first recognized as *S. cerevisiae* but were subsequently reconfirmed as *C. auris.* The WGS analysis of strains from Pakistan collected between 2012 and 2015 showed a very close relationship with minimal SNP differences to those isolated from India. Both the Pakistan and Indian strains were fluconazole resistant [21]. Dutta et al. [22] reported that a majority of the 21 *C. auris* isolated from a Bangladesh hospital exhibiting resistance to fluconazole and voriconazole.

*C. auris* infection has also been documented in healthcare facilities in West Asia. Ben-Arni et al. [23] documented the first six *C. auris* candidemia cases in Israel between May 2014 to May 2015. The fact that the isolates were found not phylogenetically related to other isolates from the East Asia, Africa, and Middle East regions, suggests that *C. auris* isolates have emerged independently in Israel. All Israel isolates were resistant to azoles but susceptible to micafungin. In Kuwait, Emara et al. [24] reported the first case of *C. auris* candidemia case in a female ICU patient with chronic renal failure. Two reports documented the first seven *C. auris* candidemia cases in Oman in 2017. All the isolates were recovered mostly from elderly patients who were admitted from August 2016 to February 2017 [25,26]. Both the Oman and Kuwait isolates were highly resistant to fluconazole. Abastabar et al. [27] described the first *C. auris* isolate in Iran which was phylogenetically distinct from other geographical isolates. Notably, this isolate was recovered from the ear canal and displayed susceptibility to all the tested antifungals, similar to the isolate from Japan [6]. Interestingly, the first *C. auris* isolate reported from the United Arab Emirates in 2017 was also susceptible to all tested antifungals [28]. Recently, Allaw et al. [29] described the first *C. auris* outbreak in a Lebanon healthcare center during COVID-19 pandemic; with all the 16 tested isolates exhibiting resistance against fluconazole and amphotericin B.

Till date, *C. auris* has been documented in four Southeast Asian countries. In Malaysia, Mohd Tap et al. [30] reported the first *C. auris* isolation from the blood of a 63-year-old neutropenic patient who eventually succumbed to the illness. Tan et al. [31] described the first three cases of *C. auris* infection in Singapore. A follow up study revealed the predominance of the South Asian clade in 71.4% *C. auris* isolated in Singapore, followed by the South American (14.3%) and East Asian (14.3%) clades [32]. Meanwhile, the first two *C. auris* isolates reported in a Thai study were fluconazole and amphotericin B resistant, and virulent as determined using a zebrafish model [33]. Xie et al. [34] described the first case of *C. auris* colonization with *C. duobushaemulonii* bloodstream infection, in a Vietnamese patient. 

**Table 1:** Chronological order of the incidences of *C. auris* infection in healthcare facilities in Asia (1996–2021).

Region (Data/Sample Collection Period)	Sample (No. of Isolates)	Noted Resistance (No. of Isolates)	Reference
South Korea (1996, 2009)	Blood (6)	Fluconazole (2)	[13]
Japan (1997–2008)	Non-blood (5)	Caspofungin (1)	[35]
		Fluconazole (1)	
South Korea (2004–2006)	Non-blood (15)	Fluconazole (7)	[36]
Japan (2009)	Non-blood (1)	None	[6]
India (2009–2011)	Blood (12)	Fluconazole (12)	[37]
India (2011–2012)	Blood (74)	Fluconazole (43)	[38]
		Amphotericin B (10)	
		Caspofungin (7)	
		Voriconazole (2)	
		Itraconazole (3)	
India (2011–2013)	Blood (7) Non-blood (8)	Fluconazole (15)	[39]
		Voriconazole (11)	
		Flucytosine (7)	
China (2011–2017)	Blood (1)	Fluconazole (15)	[16]
	Non-blood (14)		
Singapore (2012–2017)	Blood (2) Non-blood (1)	Fluconazole (3)	[31]
		Caspofungin (1)	
		Amphotericin B (2)	
Singapore (2012–2018)	Blood (4) Non-blood (3)	Fluconazole (5)	[32]
		Amphotericin (4)	
		Caspofungin (1)	
India (2013)	Blood (1)	Fluconazole (4)	[40]
	Non-blood (3)		
South Korea (2013)	Non-blood (1)	None	[41]
Kuwait (2014)	Blood (1)	Fluconazole (1)	[24]
Israel (2014–2015)	Blood (5)	Fluconazole (6)	[23]
	Non-blood (1)	Micafungin (6)	
Pakistan (2014–2015)	Blood (14) Non-blood (16)	Fluconazole (NS)	[42]
		Voriconazole (NS)	
		Anidulafungin (NS)	
		Caspofungin (NS)	
		Amphotericin B (NS)	
Pakistan (2014–2017)	Blood (75) Non-blood (118)	#Fluconazole (63)	[43]
		#Voriconazole (18)	
		#Amphotericin B (5)	
Kuwait (2014–2017)	Blood (16) Non-blood (142)	Fluconazole (56)	[44]
		Voriconazole (41)	
		Amphotericin B (13)	
		Caspofungin (1)	
		Micafungin (1)	
Kuwait (2014–2018)	Blood (58)	Fluconazole (314)	[45]
		Amphotericin B (85)	
	Non-blood (256)	Voriconazole (107)	
		Micafungin (3)	
Oman (2016–2017)	Blood (5)	Fluconazole (5)	[25]
Oman (2016–2017)	Blood (2)	Fluconazole (2)	[26]
		Amphotericin B (1)	
Kuwait (2016–2018)	Blood (7)	Fluconazole (44)	[46]
	Non-blood (42)	Amphotericin B (4)	
China (2016–2018)	Blood (8)	Fluconazole (93)	[47]
		Voriconazole (80)	
		Amphotericin B (1)	
	Non-blood (85)	Micafungin (2)	
		Anidulafungin (2)	
		Caspofungin (2)	
Oman (2016–2019)	Blood (23)	Fluconazole (23)	[48]
		Amphotericin (5)	
Japan (2017)	Non-blood (1)	None	[49]
Israel (2017)	Non-blood (4)	Fluconazole (4)	[50]
		Voriconazole (4)	
		Amphotericin B (1)	
Malaysia (2017)	Blood (1)	Amphotericin B (1)	[30]
		Anidulafungin (1)	
		Caspofungin (1)	
		Fluconazole (1)	
		Itraconazole (1)	
		Voriconazole (1)	
United Arab Emirates (2017)	Blood (1)	None	[28]
Saudi Arabia (2017–2018)	Blood (2)	Fluconazole (3)	[51]
	Non-blood (1)	Amphotericin B (2)	
Pakistan (2018)	Blood (9)	Fluconazole (14)	[52]
	Non-blood (5)	Amphotericin B (14)	
China (2018)	Non-blood (1)	None	[14]
China (2018)	Blood (2)	Fluconazole (2)	[15]
China (2018)	Blood (1)	Fluconazole (2)	[17]
	Non-blood (1)		
Kuwait (2018)	Blood (13) Non-blood (4)	Fluconazole (17)	[53]
		Voriconazole (5)	
		Amphotericin B (4)	
Taiwan (2018)	Non-blood (1)	Amphotericin B (1)	[19]
Kuwait (2018–2019)	Blood (17) Non-blood (54)	Fluconazole (55) Voriconazole (28) Itraconazole (32) Posaconazole (2) Caspofungin (32) Anidulafungin (2) Micafungin (2)	[54]
Oman (2018–2019)	Blood (11)	Fluconazole (7)	[55]
	Non-blood (21)	Amphotericin B (4)	
Saudi Arabia (2018–2019)	Blood (6)	Fluconazole (35)	[56]
		Voriconazole (35)	
	Non-blood (29)	Itraconazole (35)	
		Flucytosine (35)	
Saudi Arabia (2018–2019)	Non-blood (2)	NR	[57]
Qatar (2018–2020)	Blood (2)	#Fluconazole (11)	[58]
		#Amphotericin B (10)	
		#Caspofungin (1)	
	Non-blood (42)	#Itraconazole (1)	
		#Posaconazole (1)	
		#Voriconazole (7)	
Thailand (2018–2021)	Blood (1) Non-blood (1)	Fluconazole (2) Amphotericin (2) Itraconazole (1) Posaconazole (1) Voriconazole (1)	[33]
Saudi Arabia (2019)	Blood (2)	Fluconazole	[59]
	Non-blood (1)	Amphotericin B	
Bangladesh (2019)	Blood (14) Non-blood (7)	Amphotericin B (5)	[22]
		Fluconazole (14)	
		Voriconazole (18)	
Iran (2019)	Non-blood (1)	None	[27]
Vietnam (2019)	Non-blood (1)	Fluconazole (1)	[34]
		Amphotericin (1)	
Hongkong (2019)	Non-blood (19)	NR	[18]
Lebanon (2020)	Blood (2)	#Fluconazole (3)	[29]
	Non-blood (14)	#Amphotericin B (3)	

NR: not reported; NS: not specified; #: Not all isolates were tested. Footnote: Antifungal resistance is defined based on CLSI and EUCAST minimum inhibitory concentration (MIC) breakpoints: fluconazole, ≥32 μg/mL; voriconazole, ≥1 μg/mL; amphotericin B, ≥2 μg/mL; micafungin, ≥4 μg/mL and caspofungin, ≥2 μg/mL [44,60].

## 3. Risk Factors

According to most clinical studies in Asia, the major risk factors of *C. auris* infection include immunocompromised state of the patients, medical comorbidities or underlying chronic diseases, use of central venous and urinary catheters, prolonged stay in ICUs, and prior exposure to a variety of antimicrobials (Table 2) [38,61,62]. Almost all (11 out of 12) candidemia patients in the ICU in an Indian study were immunosuppressed due to underlying chronic diseases and had prolonged stay [37]. The stated risk factors correlated well with a *C. auris* outbreak in Venezuela, whereby all cases had previous exposure to antibiotics and had undergone invasive medical interventions prior to acquiring *C. auris* candidemia [2]. 

A retrospective analysis of candidemia reports from healthcare facilities in India [62] suggested ICU stay as one of the major predisposing factors for *C. auris* candidemia. Rudramurty et al. [38] analysed the risk factors of *C. auris* candidemia in a multicentre study of ICU-acquired candidemia in India, and showed that patients with *C. auris* candidemia had longer ICU stay prior to candidemia diagnosis. The patients who underwent invasive medical procedures during their ICU stay with central venous and urinary catheter are at a higher risk of *C. auris* candidemia than non-*auris* candidemia as the catheters provide a passage for the yeast pathogen to invade the bloodstream [61]. Furthermore, the antifungal use (fluconazole and echinocandin) prior to candidemia diagnosis were higher in *C. auris* candidemia compared with non-*auris* candidemia patients [38,63]. The occurrence of *C. auris* candidemia is notably higher in patients with prior exposure to antifungals compared to patients infected with other susceptible *Candida* species due to the exertion of selective pressure on *C. auris* by antifungal drugs [38]. However, both *C. auris* and candidemia caused by other *Candida* species displayed the same outcome both in microbiological clearance, as well as mortality rate [63]. 

A recent study showed that, *C. auris* and *Candida parapsilosis* shared almost similar habitat in the hospital environment as both *Candida* species colonize human skin and persist on medical devices and hospital surfaces [64]. Hence, *C. auris* is expected to exhibit similar risk factors as *C. parapsilosis* infection [64]. Critically ill patients are reported to acquire *C. auris* invasive infection within 48 h of admission to ICUs [65]. Recently, Shastri et al. [66] reported that patients with underlying medical conditions such as respiratory and neurological-related diseases had a greater risk of acquiring *C. auris* infection, most probably due to the prolonged stay in the ICU. Eyre et al. [67] found that the risk of *C. auris* infection/colonization was seven times greater amongst patients monitored with skin-surface temperature probes. The above findings were further proven by a subsequent study, that the act of withdrawing temperature probes following an outbreak reduced the incidence of candidemia [68].

Additionally, preterm neonates and elderly people are likely more prone to *C. auris* infections as they do not possess a strong immune system and therefore, are at a greater mortality risk to *C. auris* infection [69]. In a retrospective study, it was found that the rise in the infection/colonization rates of *C. auris* was also associated with the occurrence of diarrhea and the use of tetracycline, minocycline, and tigecycline [70]. Das et al. [71] concluded that the usage of medical equipment, antibiotics and poor hygiene practices in ICUs have led to the colonization of *C. auris* at multiple anatomical sites, leading to the occurrence of candidemia. A recent study by Sayeed et al. [64] suggested that antifungal exposure and a history of surgery are linked with a greater risk of *C. auris* infection compared to prior exposure of antibiotics, malignancy, and diabetes mellitus. 

## 4. Diagnosis

*C. auris* infections are diagnosed in routine microbiology laboratories by culturing of body fluids, blood or specimens from the affected sites [20]. 

### 4.1. Phenotypic-Based Identification

Regardless of the diagnostic methods, the accuracy of fungal pathogen identification still relies on the precision of picking *C. auris* colonies from primary culture plates. Currently, the Salt Sabouraud Dulcitol enrichment broth protocol introduced by Welsh et al. [10] has been used to isolate *C. auris* from specimens collected from clinical and environmental sources. The carbon source in the broth, dulcitol, is able to reduce the growth of other *Candida* species including *Candida glabrata* and *C. parapsilosis,* except *Candida tropicalis*. This protocol also uses 40 °C as a selective temperature for *C. auris*, as previous studies confirmed that *C. auris* can grow at 40 °C [72]. Recently, Ibrahim et al. [73] presented specific *C. auris* (SCA) medium as a diagnostic tool with higher specificity to isolate *C. auris*. The SCA medium is developed by adding crystal violet inhibitor to the initial medium developed by Welsh et al. [10] to inhibit *C. tropicalis*.

In some clinical and healthcare laboratories the fungal cultures are screened for *C. auris* colonies by plating onto CHROMagar *Candida*. The yeast pathogen is known to form beige, white, pink, and dark purple colonies on the agar. The drawback of using CHROMagar for screening is that other *Candida* species can be also recovered on the agar as they have the same morphological appearance as *C. auris* colonies [72,74].

Recently, CHROMagar^TM^ *Candida* Plus, a novel chromogenic selective medium, has been introduced for isolation of *C. auris* [75,76]. *C. auris* forms pale cream colonies at 35–37 °C, generating a distinctive blue halo surrounding the colonies after 24–48 h of aerobic incubation (https://www.chromagar.com/en/product/chromagar-candida-plus/ accessed on 12 June 2022). This selective medium helps to distinguish *C. auris* from other phylogenetically related *Candida* species, including members of the *C. haemulonii* complex, and expedites the yeast pathogen screening process [75,76].

### 4.2. Biochemical-Based Identification

Biochemical-based identification systems, for instance, Vitek 2 YST, BD Phoenix, and API 20C, have limited diagnostic capability whereby the systems often misidentify *C. auris* as other closely related *Candida* species [72]. Evaluation of the Vitek 2 (software version 8.01) showed limited ability to identify *C. auris* correctly, and in discriminating between *C. auris* and *C. duobushaemulonii* [77].

MALDI-TOF MS identifies microorganisms by comparing the unique protein profile created by the system upon receiving the input to reference databases [78]. The library databases of MALDI-TOF MS systems (Bruker-Daltonics MALDI Biotyper/bioMerieux VITEK MS) have initially included the isolates from South Korea and Japan in the database. With the increasing numbers of newly reported *C. auris* strains, updating database is necessary to improve *C. auris* identification [79]. Currently, MALDI-TOF MS is used as a rapid diagnostic tool in the clinical laboratories. The reference databases have been updated with the inclusion of all the phylogenetic clades of *Candida* species [80,81]. The advantages of this species identification system include a simple sample preparation procedure and short turnaround time [82].

### 4.3. Molecular-Based Identification

Hou et al. [83] described the use of asymmetric polymerase chain reaction (PCR) followed by molecular beacon probe-based melting curve analysis to rapidly identify mutations associated with azole and echinocandin resistance in *C. auris*. The substitutional mutations in the *ERG11* locus (Y132F and K143R) and *FKS1* locus (S639F) were used as the markers to detect clinical *C. auris* isolates with high accuracy without sequencing. 

A loop-mediated isothermal amplification (LAMP)-based method was developed by Yamamoto et al. [84] for rapid identification of *C. auris*. The assay, targeting the pyruvate: ferredoxin oxidoreductase domain region of *C. auris* genome, has a short turnaround time, and is able to discriminate *C. auris* from closely related species and other fungi. This assay enables early diagnosis as it allows direct detection of *C. auris* from clinical specimen.

Leach et al. [85] developed a rapid real-time PCR assay using TaqMan probe. The assay is able to process a large number of surveillance samples and is highly specific and reproducible. It is highly sensitive as it targets and amplifies the multicopy internal transcribed spacer 2 (ITS2) regions of the ribosomal gene. Similarly, SYBR green *C. auris* quantitative PCR (qPCR) assay has also been developed and validated using skin swabs [86]. Molecular assays such as PCR amplification or real-time PCR assays reduce the workload of clinical laboratories in the processing of swab samples, as only a small volume of sample is required for direct DNA isolation, and can be used to detect both dead and living cells [72]. 

Accurate and reliable identification of *C. auris* has been achieved by sequence determination of *C. auris* ITS region or the D1/D2 domains of rDNA [76]. The importance of sequencing method was highlighted by Ninan et al. [87] in view of the possibility of misidentifying *C. auris* in clinical laboratories.

In a nutshell, rDNA sequencing and MALDI-TOF MS have been regarded as the most reliable, rapid, and efficient approaches in the identification of *C. auris*, however; the equipment required is not available for every clinical laboratory due to high cost and technical demands [88]. Molecular-based assays can be used for routine identification of fungal species in clinical laboratories since reliable results can be obtained within several hours [72].

## 5. Colonization and Transmission

*C. auris* was recovered mostly from hospital environments, especially moist surfaces. This indicates that contaminated healthcare surfaces can be the potential source of transmission [89,90]. According to Rossato et al. [3], the ability of *C. auris* to form cellular aggregates, as well as its tolerance towards high salinity and temperatures of up to 42 °C promote its persistence in the hospital environment.

Patterson et al. [91] described a small *C. auris* outbreak in ICUs where the cloth lanyard holding a key to access controlled medications often used by nursing staff in two ICUs was the source of *C. auris* infection. The yeast pathogen adhered and persisted for at least two weeks on the lanyard as it is made of polyester or nylon type of material. *C. auris* has been recovered from non-medical objects including curtains, floors, windows, and bed rails [91]. However, their role in transmission is still unproven.z

Previous studies have revealed that *C. auris* isolation from non-sterile body sites more likely represents colonization rather than infection [92,93]. Das et al. [94] observed *C. auris* colonization at different anatomical sites of the body, for instance, axilla, tracheostomy, and groin, due to continuous use of intravenous broad-spectrum antibiotics, prolonged ICU stay, excessive use of medical device and poor surveillance strategies. The colonization of *C. auris* in these anatomical sites can be one of the factors causing infection in the bloodstream. *C. auris* colonization on patients’ skin and also other parts of the body may have facilitated the horizontal transmission of the pathogen to other patients via shedding and persistence in the healthcare environment [95].

Additionally, *C. auris* can stay alive and continue to persist on abiotic surfaces, such as steel and plastic objects for months [10,96]. Piedrahita et al. [4] reported that *Candida* species including *C. auris* have comparable survival rates on both dry and moist surfaces [89,97]. 

A comparative analysis of the pathogenicity of 12 *C. auris* isolates from the UK using the invertebrate model, *Galleria melonella,* revealed two types of *C. auris* strains, which are the aggregate-forming strain and non-aggregate-forming strain [98,99]. The aggregating strain is less virulent, and has better survival capacity, exhibits selective tolerance to biocides and unique ability to transfer to new and sterile surfaces after treatment, as compared to the non-aggregate-forming strain [96].

As soon as *C. auris* colonizes healthcare environment, it activates the stress-activated protein kinase Hog1 for its adaptation to the environment and maintenance of its cell phenotype [100]. During this transition, phospholipase and proteinase are secreted by *C. auris* in a strain-dependent manner, to aid its pathogenesis [101]. First, the hydrolytic enzymes activate glycosylphosphatidylinositol (GPI)-anchored cell wall proteins and adhesins for adherence of biofilms on healthcare surfaces and medical devices via nonspecific interactions such as specific adhesin–ligand bonds [99,100]. Phospholipases play an important role in the pathogenesis by damaging the host cell and evading the host immune system [97,102]. Second, through the production of extracellular matrix via the expression of KRE6 and EXG genes in biofilms, *C. auris* acquires resistance to osmotic stress and disinfectants usually used in healthcare facilities. As a result, C. *auris* may infect the skin of the healthcare staff and patients in ICUs via direct contact with the contaminated surfaces [100]. Third, *C. auris* secretes haemolysins to facilitate iron assimilation from the haemoglobin--heme group for enhancement of its survival within the host [97,103]. These virulence factors may play essential roles in the rapid spreading of *C. auris* in healthcare settings.

The higher activity of *C. auris* secreted aspartyl proteinase (SAP) at 42 °C indicates that the yeast pathogen is able to maintain its pathogenicity even at elevated temperatures [97]. Furthermore, the investigation of an outbreak in Colombia revealed the linkage among patients, healthcare workers, and the environment for the transmission of *C. auris*. Strains that were isolated from healthcare workers are genetically identical to the strains recovered from the infected patients and also the environment [93,104]. Furthermore, the robust biofilm production of *C. auris* has been linked with implant-associated infections, for instance bloodstream infection, central nervous system infection, and prosthetic joint infection [105]. *C. auris* isolated from catheter-associated candidemia of a rodent model displayed adherence and proliferation as biofilms composed of yeast cells on catheter surfaces [105,106]. 

Interestingly, according to Misseri et al. [107], the emergence of *C. auris* and its transmissibility to humans may have association with global warming. The effect of climatic oscillations on wetlands possibly contributes to the thermal and salinity tolerance of *C. auris*. The virulence factors contributing to the persistence of *C. auris* in the ecosystem may be acquired through induced genetic mutation possibly through a combined effects of global warming and UV radiation. In a nutshell, the fact that *C. auris* is able to persist on medical devices for an extended period and poses a high infectious risk, explains for the high transmission rates among patients and healthcare workers in clinical settings. 

## 6. Prevention and Infection Control Measures

As *C. auris* is emerging and persisting in healthcare facilities, it is of utmost important to implement adequate infection prevention and control (IPC) procedures and screening protocols to control infection and transmission. Currently, there is no standardized environmental cleaning or disinfection method to control nosocomial transmission of *C. auris* at the healthcare facilities. At present, some healthcare settings use quaternary ammonium compounds such as Lysol and Virex II 256 for disinfection, however; ineffectiveness against *C. auris* has been reported [61,108].

Several health organizations have recommended a variety of cleaning or disinfection methods using disinfectants and cleaning agents. The CDC recommends that colonized or infected patients in hospitals are quarantined in a private room and active cleaning with an efficient Environmental Protection Agency (EPA)-registered hospital-grade disinfectant is required to disinfect the contaminated surfaces [108,109]. Similarly, Public Health England (PHE) suggests the use of hypochlorite (1000 ppm)-based products for environmental cleaning. Meanwhile, disinfectants with certified antifungal activity are recommended by the European Center for Disease Prevention and Control (ECDC) for terminal cleaning. Some health organizations also recommend regular cleaning in addition to terminal cleaning using a chlorine (1000 ppm)-based disinfectant agent [108].

Previous studies have shown the effectiveness of chlorine-based disinfectants against *C. auris* [110]. Additionally, different concentrations of chlorine-based disinfectants displayed similar effectiveness in killing *C. auris* on surfaces such as cellulose matrix, stainless steel, and polyester within 5 and 10 min of contact time [111]. Exposure to hydrogen peroxide vapor has shown 96.6–100% elimination of *C. auris* isolates [112]. However, the efficiency rate varies depending on the concentrations of vapor. A 1.4% hydrogen peroxide disinfectant requires a shorter time of contact, which is 1 min, whereas a 0.5% hydrogen peroxide disinfectant needs at least 10 min for effective killing [108]. Meanwhile, the World Health Organization (WHO) suggests disinfection using 0.1% bleach after cleaning with soap and water [108]. Furthermore, some authors suggested the use of UltraViolet-C (UV-C) light for terminal decontamination, which requires at least 20 min of exposure [108,113].

In some healthcare settings, chlorhexidine gluconate (CHG) is frequently used for skin decontamination. CHG with a concentration of less than 0.02% has been found to inhibit the growth of *C. auris* biofilms effectively within a 24 h-contact time [114]. Previously, the rapid growth inhibition of *C. auris* isolates within a shorter contact time (i.e., 3 min), using a range of CHG concentration between 0.125% and 1.5%, has been reported by Abdolrasouli et al. [112]. The efficiency of CHG was further proven during an outbreak in a Spanish tertiary care center, where 2% aqueous chlorhexidine wipes were used for daily skin decolonization of patients to reduce the transmission of *C. auris* [115]. Meanwhile, Moore et al. [110] reported that a mixture of 70% isopropyl alcohol and 2% CHG was more effective in eliminating *C. auris* isolates within a 2 min-contact time compared to 2% CHG alone. Regardless of the method, Kenters et al. [116] suggested that the frequency of cleaning and disinfection in intensive care settings should be at least twice every day.

In addition, the experiment conducted by Heaney et al. [117] suggested that imposing heat shocks as an additional process during the hospital laundry process together with the usage of alcohol ethoxylate, thermal, and alkaline stress would promote the killing of the fungal pathogen. Lamoth et al. [118] suggested systematic screening of multiple anatomic sites including axilla, nares, and groins, for patients coming from regions with a high prevalence of *C. auris* infection, as these sites might be highly colonized by the fungal pathogen. Together with rapid detection of cases and potential reservoirs, these strategies may assist in the prevention of C. *auris* outbreaks in healthcare facilities. Kenters et al. [116] recommended a weekly screening as the fungal pathogen possibly resurfaces after extensive medical interventions. In addition, the authors also recommended the use of disposable biomedical products and equipment in healthcare facilities and no sharing of items with other wards or ICUs, as reusable items can increase the risk of transmission.

In order to prevent fungal colonization on the skin and transmission from patients to healthcare workers, healthcare workers should wear proper personal protective equipment (PPE), gloves, and surgical masks when entering *C. auris*-positive patient’s room [116]. Additionally, healthcare workers and personnel should practice frequent hand washing using soap and water or scrubbing using alcohol-based hand sanitizer [61].

In a nutshell, implementation of strict intervention and effective prevention control measures are necessary to stop *C. auris* transmission in healthcare facilities. This includes the quarantine of patients and their close contacts, use of personal protective clothing and hand hygiene by healthcare professionals, culture and molecular surveillance of patients and environmental surfaces, regular and terminal cleaning of healthcare facilities, and decontamination of the skin using appropriate disinfectants and antiseptics.

## 7. Conclusions

The recent emergence of *C. auris* as one of the leading causes of invasive fungal infections has garnered the attention of the scientific and healthcare community. The yeast pathogen has posed a major threat to the medical field, as major outbreaks have occurred amongst patients with medical comorbidities and resulted in high rates of mortality. *C. auris* infection has been a challenge for treatment and management due to its poor detection by clinical diagnostic methods, rapid dissemination, and reduced susceptibility to disinfectants and multiple antifungal drugs. The detection of possible risk factors, identification of reliable diagnostic methods that facilitate early diagnosis and therapy, and implementation of effective preventive and control strategies of *C. auris* may reduce the incidences of possible invasive fungal infections in healthcare facilities.

## Figures and Tables

**Table 2:** List of risk factors of *C. auris* infections based on some published studies from Asia (1996–2021).

Region	Duration of Data/ Sample Collection	Total Number of Patients (No of Candidemia Patients)	Mortality (%)	Risk Factors	Reference
South Korea	1996, 2009	3 (3)	66.67	Presence of CVC, prior antibiotics exposure, surgery, ICU admission, indwelling urinary catheter	[13]
Japan	2009	1 (0)	0	History of chronic otitis media and type 1 diabetes mellitus	[6]
India	2009–2011	12 (12)	50	Indwelling urinary catheter, prior antimicrobial drug exposure, presence of CVC, ICU admission, cancer chemotherapy, HIV, diabetes mellitus, chronic kidney disease	[37]
India	2011–2012	74 (74)	5.3	Prolonged ICU stay, underlying respiratory illness, vascular surgery, prior antifungal exposure	[38]
India	2011–2013	12 (7)	33.3	Usage of urinary catheter, prior antibiotics exposure, surgery, ICU admission	[39]
China	2011–2017	15 (1)	NR	Diarrhea, prior antibiotics exposure	[16]
Singapore	2012–2017	3 (2)	33.33	Underlying medical conditions, ICU admission, prior antifungal exposure	[31]
India	2013	1 (0)	100	Underlying medical conditions, prior antibiotics exposure	[40]
Kuwait	2014	1 (1)	100	ICU admission, prior antimicrobial drug exposure	[24]
Pakistan	2014–2015	30 (14)	53.3	Surgery, chronic kidney disease, ICU admission, urinary catheter, presence of CVC	[42]
Pakistan	2014–2017	92 (38)	42.4	Surgery, prior antibiotics exposure, ICU admission	[43]
Kuwait	2014–2017	56 (13)	NR	ICU admission, underlying medical conditions, prolonged hospital stay	[44]
Oman	2016–2017	5 (5)	60	ICU admission, presence of CVC, prior antibiotic therapy	[25]
Oman	2016-2017	2 (2)	50	ICU admission, diabetes mellitus, chronic kidney disease, presence of CVC	[26]
Kuwait	2016–2018	18 (7)	55.6	Underlying medical conditions, surgery, prior antifungal exposure, indwelling urinary catheter	[46]
Japan	2017	1 (0)	0	Underlying medical conditions	[49]
Israel	2017	2 (0)	NR	Presence of CVC	[50]
Malaysia	2017	1 (1)	100	Neutropenia condition, prolonged hospital stay, prior antibiotics exposure, presence of CVC	[30]
United Arab Emirates	2017	1 (1)	100	Underlying medical conditions, prolonged hospital stay, ICU admission	[28]
Saudi Arabia	2017–2018	3 (2)	33.33	Underlying medical conditions, presence of CVC, surgery	[51]
Pakistan	2018	14 (9)	42.9	Surgery, presence of CVC, usage of urinary catheters, prior antifungal exposure	[52]
Kuwait	2018	17 (13)	60	Underlying medical conditions, prolonged hospital stay	[53]
Kuwait	2018–2019	71 (17)	52.1	Underlying medical conditions, hospital stay, ICU admission	[54]
Oman	2018–2019	32 (11)	53.1	Underlying medical conditions, prior antifungal exposure	[55]
Saudi Arabia	2018–2019	34 (6)	20	Underlying medical conditions, ICU admission, prolonged ICU stay, prior antifungal exposure, surgery, presence of CVC, indwelling urinary catheter	[56]
Qatar	(2018–2020)	36 (2)	NR	ICU admission, surgery, underlying medical conditions	[58]
Lebanon	2020	14 (3)	35.7	ICU admission, presence of CVC, indwelling urinary catheter, prior antifungal exposure	[29]

CVC: central venous catheter; NR: not reported.
